# Biomarkers for Amyotrophic Lateral Sclerosis and Frontotemporal Dementia Associated With Hexanucleotide Expansion Mutations in *C9orf72*

**DOI:** 10.3389/fneur.2018.01063

**Published:** 2018-12-05

**Authors:** Mary Kay Floeter, Tania F. Gendron

**Affiliations:** ^1^National Institute of Neurological Disorders and Stroke, National Institutes of Health, Bethesda, MD, United States; ^2^Department of Neuroscience, Mayo Clinic, Jacksonville, FL, United States

**Keywords:** *C9orf72*, cortical thinning, diffusion tensor imaging, dipeptide repeat proteins, functional connectivity, motor neuron disease, neurofilament proteins, biomarker

## Abstract

Now that genetic testing can identify persons at risk for developing amyotrophic lateral sclerosis (ALS) many decades before symptoms begin, there is a critical need for biomarkers that signal the onset and progression of degeneration. The search for candidate disease biomarkers in patients with mutations in the gene *C9orf72* has included imaging, physiology, and biofluid measurements. In cross-sectional imaging studies, C9+ ALS patients display diffuse reductions of gray and white matter integrity compared to ALS patients without mutations. This structural imaging signature overlaps with frontotemporal dementia (FTD), reflecting the frequent co-occurrence of cognitive impairment, even frank FTD, in C9+ ALS patients. Changes in functional connectivity occur as critical components of the networks associated with cognition and behavior degenerate. In presymptomatic C9+carriers, subtle differences in volumes of subcortical structures and functional connectivity can be detected, often decades before the typical family age of symptom onset. Dipeptide repeat proteins produced by the repeat expansion mutation are also measurable in the cerebrospinal fluid (CSF) of presymptomatic gene carriers, possibly throughout their lives. In contrast, a rise in the level of neurofilament proteins in the CSF appears to presage the onset of degeneration in presymptomatic carriers in one longitudinal study. Cross-sectional studies indicate that neurofilament protein levels may provide prognostic information for survival in C9+ ALS patients. Longitudinal studies will be needed to validate the candidate biomarkers discussed here. Understanding how these candidate biomarkers change over time is critical if they are to be used in future therapeutic decisions.

## Introduction

A repeat expansion mutation in the *C9orf72* gene is the most common cause of familial amyotrophic lateral sclerosis (ALS) in people of Northern European ancestry and accounts for 5-10% of sporadic ALS cases in Europe and the USA (1, 2). The *C9orf72* mutation (C9+) is also a common cause of familial frontotemporal dementia (FTD) (3). The clinical phenotype is often mixed, and many C9+ ALS patients have some degree of cognitive impairment, ranging from mild executive dysfunction to frank FTD (4). Because mutation carriers can be identified by genetic testing many decades before symptoms begin, there is considerable interest in biomarkers to identify when degeneration begins and to monitor disease progression. Currently, development of such biomarkers is at the early stage of identifying measures that differ in group comparisons. This review will discuss the current status of studies of non-invasive biomarkers such as imaging and physiology, and minimally invasive biomarkers derived from biofluids.

## Imaging Studies

There is particular interest in neuroimaging as a biomarker because it offers a way to visualize pathological changes in the brains of living patients. In autopsy studies, brains from C9+ patients exhibited the neuronal loss, gliosis, and TDP-43 inclusions characteristic of sporadic ALS and some FTD patients (5), as well as the nuclear RNA foci and cytoplasmic aggregates of dipeptide repeat (DPR) proteins specific to the *C9orf72* mutation (5, 6). The distribution of these pathologic findings differs between C9+ ALS and C9+ FTD patient brains (7, 8). The story emerging from neuroimaging studies is that the diversity of clinical phenotypes reflects the extent to which the most affected brain regions contribute to networks that underlie cognitive, behavioral, motor, and language function (9, 10).

### Structural MRI—Gray Matter Atrophy

In structural MRI scans, C9+ ALS patients displayed extensive, relatively symmetric volume loss and cortical thinning compared to similarly aged healthy subjects (1, 11–14). Compared to C9– ALS patients (i.e., without the *C9orf72* mutation), C9+ ALS patients had greater atrophy of extra-motor cortical regions, particularly parieto-occipital cortical areas, including the cuneus and precuneus (11–13), and relatively less atrophy of the precentral motor cortex (13, 14). Correlations between volumetric changes and cognitive testing measures have led several investigators to conclude that the predominant gray matter imaging pattern in C9+ ALS patients is associated with cognitive changes (11–14). A similar pattern of diffuse, relatively symmetric cortical volume loss is found in C9+ FTD patients (15–19).

Several studies report more atrophy of subcortical structures in C9+ ALS than in C9– ALS patients. The topographic specificity of connections between these subcortical structures and specific cortical regions can lead to discrete functional deficits. Nearly all volumetric studies to date have reported thalamic atrophy in C9+ carriers. Thalamic atrophy has been reported in C9+ ALS patients (11–13), C9+ FTD patients (15, 16, 18–22), and presymptomatic C9+ carriers (23–26). Although C9+ ALS patients may have more thalamic atrophy compared to C9– ALS patients with a similar degree of cognitive impairment (11), the association between thalamic atrophy and cognitive impairment can be seen in FTD patients with other gene mutations (27) and C9– ALS patients with cognitive impairment (28). Because there is topographic specificity of corticothalamic circuits, degeneration of particular thalamic nuclei should produce different functional impairments. However, most MRI studies measured the hemi-thalamus in its entirety. Using a more refined segmentation scheme in a cohort of C9+ FTD patients, Lee and colleagues (20) found atrophy specifically in the medial pulvinar nucleus of the thalamus, a multisensory nucleus with connections to posterior parietal, prefrontal, and cingulate cortical areas (29). Schonecker and colleagues reported greater atrophy of motor sub-regions of the thalamus in symptomatic C9+ carriers (30).

Atrophy of other subcortical structures has also been reported. The cerebellum has been of particular interest because high levels of DPR proteins (8, 31, 32) and RNA foci were found in cerebellar Purkinje and granule cells in C9+ patients (33), and levels of cerebellar DPR proteins in C9+ ALS were correlated with cognitive impairment (31). While a pathological study reported no appreciable neuronal loss in the cerebellum (15), cerebellar atrophy has been reported in some, but not all, imaging studies. Detection differences largely reflect whether the whole cerebellum or focal cerebellar regions were measured. Changes in focal cerebellar regions, such as in lobule VIIa/crus I, were found in several studies of C9+ ALS and C9+ FTD patients (11, 17, 21, 27, 34). This region of the cerebellum has been mapped in functional MRI studies to cortical association networks, including the dorsolateral prefrontal cortex and parietal association areas that play a role in executive function (35). Volume loss has also been reported in various nuclei of the basal ganglia in C9+ ALS and C9+ FTD patients (20, 28, 36), a finding associated with cognitive and behavioral scores across the spectrum of ALS and FTD, and thought to result from disruption of corticostriatal circuits (37). Two studies also reported hippocampal atrophy in C9+ ALS (11, 38), a finding consistent with the occurrence of hippocampal sclerosis in some C9+ ALS-FTD brains (5) and memory deficits.

**Figure 1:**
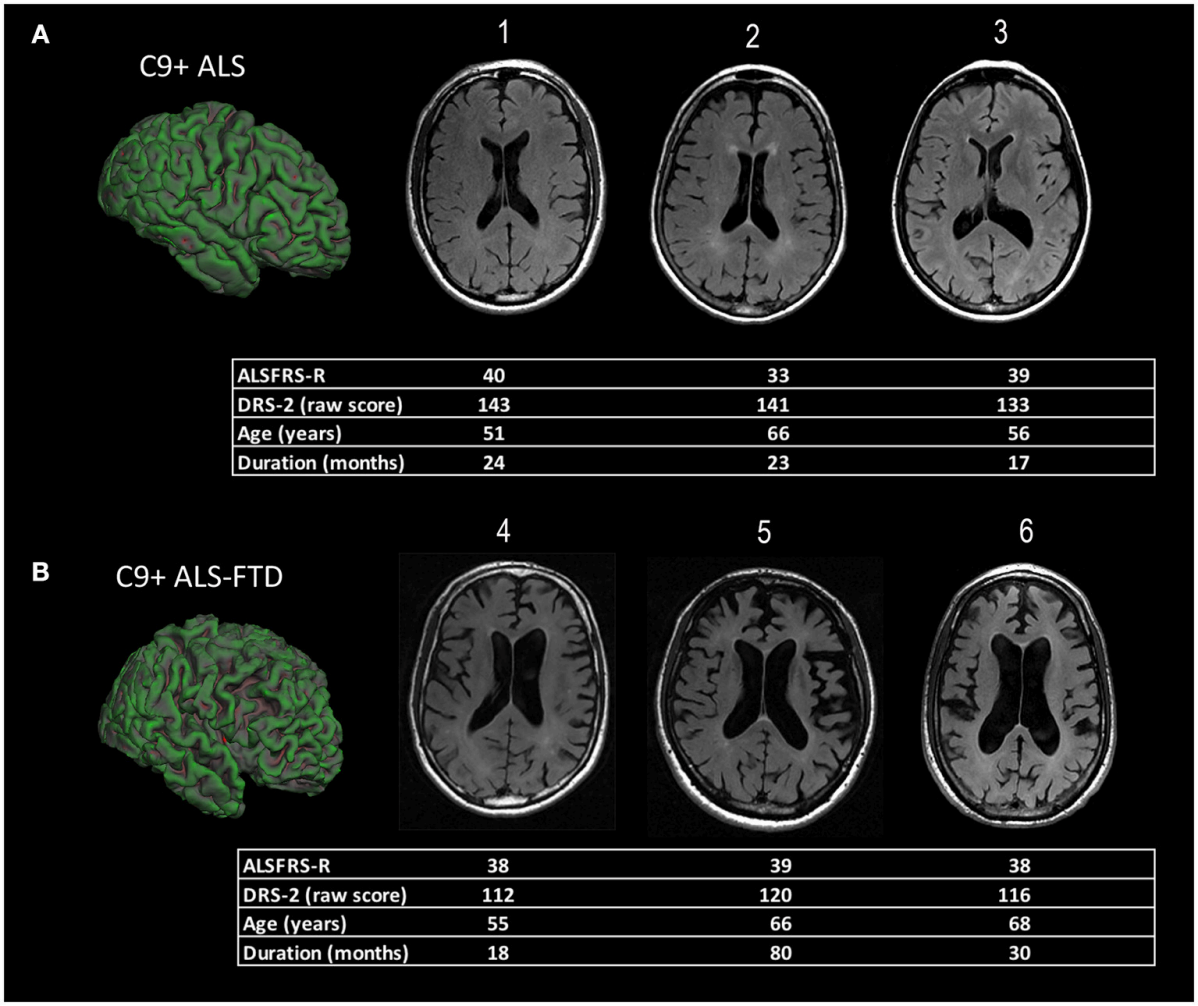
Representative examples of diffuse cortical atrophy in MRI scans of ALS patients with *C9orf72* mutations. The demographic information and scores on motor and cognitive scales are listed below each patient's scan. **(A)** Compared to age-matched controls, mild ventricular enlargement was seen in C9+ patients 1, 2, and 3 who had ALS, but good cognitive function, as evidenced by their scores on the Mattis Dementia Rating Scale−2 (DRS-2). The surface rendering of one patient [left side of panel **(A)**] shows sulci in frontal lobe sulci are also mildly enlarged compared to the occipital lobe. **(B)** C9+ patients 4, 5, and 6 had ALS-FTD with a similar degree of motor dysfunction to those in panel **(A)**, as measured by their ALS functional rating scale revised (ALSFRS-R) scores, but marked cognitive impairment with low DRS-2 scores. There is marked enlargement of ventricles evident in axial slices, as well as enlargement of frontal and temporal sulci in the surface rendering at left of panel **(B)**.

The diffuse nature of the brain atrophy, involving cortical and subcortical structures, has led to the suggestion that changes in ventricular volume be used to follow longitudinal disease progression in C9+ carriers (13, 17, 34) (Figure 1).

#### Pathological Correlates

The distribution of atrophy in structural MRI scans of C9+ ALS and FTD patients mirrors the distribution of neuronal loss and TDP-43 pathology in brains of C9+ ALS-FTD patients (5) and sporadic ALS and FTD patients (39). However, the relationship between these hallmarks of degeneration–neuronal loss, gliosis, and TDP-43 inclusions—and the RNA foci and DPR protein aggregates specific for the C9+ genotype is still evolving. Unlike TDP-43 pathology, which closely parallels neurodegeneration, the distribution of RNA foci (33) and DPR protein pathology do not (6–8, 33, 40, 41), although reports on the latter have been somewhat conflicting. A moderate association between the amount of poly(GA) dystrophic neurites and degeneration in the frontal cortex was observed (40), and inclusions of poly(GR), which is especially toxic in *in vitro* models (42), correlated with TDP-43 pathology and neurodegeneration in C9+ FTD-ALS brains (7, 41). Nevertheless, the presence of DPR protein aggregates and RNA foci did not lead to TDP-43 accumulation in a neurologically healthy mosaic carrier (43), and DPR protein pathology with little, if any, TDP-43 pathology was observed in a c9FTD kindred with early intellectual disability (44) and three *C9orf72* mutation carriers who developed relatively rapid cognitive decline but died prematurely due to unrelated illness (45).

### Diffusion Tensor Imaging of White Matter Tracts

In diffusion tensor imaging (DTI) studies, C9+ ALS patients showed more widespread loss of white matter integrity compared to healthy controls and C9– ALS patients, most commonly in the frontal white matter, as measured by decreased fractional anisotropy, increased radial diffusivity, or increased mean diffusivity (11, 12, 14, 38, 46). Several white matter tracts affected in C9+ ALS are not typically affected in cognitively intact C9– ALS patients, including the genu of the corpus callosum, anterior limbs of the internal capsule, thalamic radiations, and long association tracts such as the uncinate fasciculus, superior longitudinal fasciculus, and inferior longitudinal fasciculus (11, 12, 14, 38, 46). These frontal and association tracts were also affected in diffusion studies of C9+ FTD patients (17, 20, 36), and presymptomatic C9+ carriers in some studies (47). Motor tracts, including the corticospinal tract and motor segment of the corpus callosum, were affected in C9+ ALS patients compared to healthy controls (11, 46), but exhibited less disruption than in C9– ALS patients (14). In a group of C9+ carriers with a mixture of phenotypes, changes in diffusion indices of specific tracts correlated with clinical measures: frontal white matter correlated with lexical fluency and behavioral scores, and changes in motor tracts correlated with the ALS functional rating scale (46).

### Unresolved Questions About Structural Imaging as a Biomarker

Several questions arise from the findings in structural MRI scans. First, does a genotype-specific C9+ MRI signature exist? To address this question, Westeneng and colleagues (38) identified a candidate “genotype-specific MRI signature” in a model comparing 92 volumetric and DTI variables in scans of 28 C9+ to 28 C9– ALS patients. Although 11 imaging variables identified a C9+ specific signature in the training dataset, nearly 20% of C9– ALS patients in a large validation dataset were classified as having the C9+ MRI signature. Misclassified patients scored more poorly on a measure of executive function, thus underscoring the close association between neuroanatomical atrophy patterns and clinical phenotypes. A second question is whether the volumetric differences in adult C9+ carriers arise during development or are a consequence of degeneration. This question was addressed in imaging studies comparing relatively young presymptomatic C9+ carriers (< age 40) to non-carriers from the same families. Although older presymptomatic C9+ carriers had clear evidence of atrophy compared to similarly-aged C9– family members, so did younger C9+ presymptomatic carriers when compared to C9– family members of the same age (24–26, 47, 48). Cortical and subcortical structures were smaller, particularly the thalamus, in younger C9+ carriers. The common genetic background of family members with and without the *C9orf72* mutation facilitated detection of small differences in these studies. Lee and colleagues found that smaller gray matter volumes occurred across a range of ages in presymptomatic C9+ carriers and had a similar age-related decline as in C9– controls, suggesting a developmental origin (47). Longitudinal studies in individual C9+ carriers before and after the onset of symptoms will be needed to truly determine whether congenitally small brain structures begin accelerated volume loss with the onset of degeneration in adulthood or whether the *C9orf72* mutation leads to slow, lifelong accumulation of subclinical pathology. Lastly, because the distribution of atrophy mirrors the distribution of TDP-43 in pathological studies (5), longitudinal structural imaging, in combination with clinical phenotyping, can be used to test hypotheses that TDP-43 pathology spreads through axonal connections. Pathological studies in sporadic ALS have led to the proposal that TDP-43 pathology spreads through corticofugal projections (49). In contrast, in behavioral-variant FTD, TDP-43 pathology has been proposed to spread from orbitofrontal cortex to posterior regions through axonal tracts (50).

### Functional Connectivity

Changes in functional connectivity using task-based or resting state fMRI have been reported prior to development of clinical symptoms in patients with *GRN* or *MAPT* mutations at risk for FTD (51). Three studies examined changes in functional connectivity in resting state networks in C9+ carriers. One study in symptomatic carriers found that C9+ and C9– behavioral variant FTD patients had disruption of salience network connectivity that was associated with neuropsychiatric severity, as well as disruption of sensorimotor connectivity (20). The disruption of the salience network occurred with atrophy of different nodes within the salience network in individual patients (20). Disruption of the salience network and a network generated from a medial pulvinar nucleus seed was also observed in young presymptomatic C9+ carriers (47). Another study reported increased connectivity in the visual network of C9+ carriers with a mixture of motor and cognitive phenotypes compared to sporadic cases with similar phenotypes (11).

### Proton Emission Tomography

Hypometabolism in the frontal lobes in FDG-PET studies is considered supportive of a clinical diagnosis of FTD (52). The few reports of PET imaging in C9+ carriers had slightly different findings. In one study, C9+ ALS patients had more widespread hypometabolism occurring in the cingulate, insula, caudate, and thalamus, with clusters of hypermetabolism in occipital, left precentral, left postcentral, and superior temporal cortex when compared to C9– ALS patients with or without FTD (53). In contrast, the other study reported that C9+ ALS and C9– ALS patients exhibited hypometabolism in peri-rolandic cortex; several prefrontal regions had hypometabolism in both groups, but C9+ ALS patients alone had focal hypometabolism in the thalamus and posterior cingulate cortex (54). One case study also reported frontal and temporal hypometabolism in a C9+ ALS patient who subsequently developed FTD (55). Another reported that amyloid imaging, but not FDG-PET, distinguished FTD from Alzheimer disease in a C9+ carrier (56).

## Physiology

Physiological methods have been used to assess cortical function in C9+ carriers. Transcranial magnetic stimulation (TMS) is a non-invasive technique for assessing cortical excitability. Numerous TMS studies in sporadic ALS patients have provided evidence for hyperexcitability of the motor cortex early in disease (57), with loss of excitability at late stages (58). C9+ ALS patients were similarly found to have increased cortical excitability according to several different TMS indices, but presymptomatic C9+ carriers did not (59–61). Evoked potential measures have been used to explore particular cognitive functions in C9+ patients (62), but have not been routinely used to identify disease onset or severity. Electroimpedance myography (63) and Motor Unit Number Index (MUNIX) (64) are non-invasive methods that have been used to follow lower motor neuron dysfunction in ALS patients in clinical trials but, to date, have not been reported in C9+ ALS patients.

## Energy Metabolism

Patients with ALS develop defects in energy metabolism that include low body mass index (BMI), hypermetabolism, and hyperlipidemia (65, 66). While the contribution of dysregulated energy homeostasis to ALS pathogenesis remains to be resolved, such defects correlate with prognostic factors. For instance, weight loss and hypermetabolism are associated with faster disease progression and shorter survival in ALS (66–68). The cause of these metabolic changes is unknown, but may result from hypothalamic atrophy. Gorges et al. (69) have shown that the hypothalamus is atrophied in ALS patients and in presymptomatic ALS mutation carriers (the latter were comprised predominantly of C9+ individuals). Furthermore, they found a modest but significant correlation between hypothalamic volume and BMI, especially in patients with familial ALS, and observed that anterior hypothalamic volumes correlate with age of disease onset (69). While these findings are not specific to C9+ carriers, they do suggest that hypothalamic atrophy, BMI, and disturbances in energy homeostasis could provide prognostic insight.

## CSF and Biofluid Studies

Fluid-based biomarker discovery efforts for ALS have most often been conducted using cerebrospinal fluid (CSF) due to its proximity to affected neuroanatomical regions. However, progress has been made using plasma and serum, and studies using urine and saliva are emerging (70). Among the more widely studied biomarker candidates are inflammatory mediators, metabolic markers and neurofilament proteins; the latter, however, have arguably garnered the most attention (70, 71). Neurofilament proteins, which include neurofilament heavy chain (NfH), neurofilament medium chain and neurofilament light chain (NfL), are abundantly and exclusively expressed in neurons where they form the neuronal cytoskeleton. Because neurofilament proteins are released from neurons upon axonal damage or degeneration, they are considered indicators of neuronal injury for various neurological disorders.

### Neurofilament Proteins

In C9+ carriers, levels of CSF phosphorylated NfH (pNfH) were significantly higher in patients with ALS or FTD compared to asymptomatic individuals, and strongly associated with survival in patients with C9+ ALS (72). Notably, C9+ ALS patients had significantly higher pNFH levels than C9– ALS patients, which presumably reflected increased neurodegeneration, consistent with reports that patients with C9+ ALS develop greater brain atrophy, particularly in extra-motor regions, compared to C9– ALS patients (11–13). More diffuse degeneration may account for the shorter survival of C9+ ALS patients compared to C9– ALS patients (1, 72–75). Similar to pNfH, CSF NfL levels were elevated in symptomatic compared to presymptomatic C9+ carriers (76, 77), and higher NfL levels in symptomatic individuals correlated with greater disease severity and shorter survival (77). Furthermore, elevated CSF NfL in C9+ carriers was associated with lower gray matter volumes in the ventral and dorsomedial prefrontal cortex, ventral, and dorsal insula, anterior cingulate, caudate, medial thalamus, and other frontotemporoparietal regions (77).

These findings supporting CSF pNfH and NfL as prognostic markers for C9+ patients could significantly impact drug development. For instance, the heterogeneity of disease course in C9+ ALS could result in different proportions of fast and slow progressors in clinical treatment arms. Using pNfH and NfL levels as surrogates for progression rate could facilitate stratification of patients into balanced groups to reduce variability in treatment outcomes. Early evidence also suggests that NfL in CSF and serum can inform the potential phenoconversion of individuals from an asymptomatic to a symptomatic state (78). Through the study of individuals that carry a mutation in *C9orf72* or other ALS-associated genes, Benatar and colleagues found that NfL in asymptomatic mutation carriers was elevated above the range seen in healthy individuals as early as 12 months prior to the earliest clinical symptoms (78). Should these findings be validated in additional cohorts, NfL could provide insight on when neurodegeneration begins. This would facilitate the timely diagnosis of C9+ ALS, and increase the likelihood of enrolling patients in clinical trials at an early stage of disease when they are most likely to benefit from therapeutic intervention.

### Dipeptide Repeat Proteins

In addition to prognostic biomarkers, markers of target engagement would improve the interpretation of clinical trials for C9+ ALS and FTD. As mentioned above, a characteristic neuropathological feature of C9+ ALS and FTD is the presence of neuronal inclusions formed of DPR proteins synthesized from expanded *C9orf72* repeats. One of these proteins, poly(GP), is abundantly expressed in the brain of C9+ carriers and is detected in CSF (72, 77, 79, 80). While several studies observed that CSF poly(GP) did not associate with age at disease onset, survival, or markers of neurodegeneration (e.g., CSF pNfH or NfL, or measures of brain atrophy) (72, 77, 79), poly(GP) shows promise as a pharmacodynamic biomarker (81).

Since RNA transcripts of expanded *C9orf72* repeats are believed to play a key role in C9+ ALS and FTD (82), therapeutic strategies that target *C9orf72* repeat RNA are being developed. Given that levels of poly(GP) correlated with levels of repeat-containing RNA in the cerebellum of C9+ carriers (31, 83), poly(GP) was investigated as a marker of target engagement for repeat RNA-based therapies. Antisense oligonucleotides (ASOs), small molecules and genetic modifiers that target *C9orf72* repeat RNA attenuated poly(GP) levels in various preclinical models including yeast, worms, mice, and C9+ ALS patient cell lines (81, 84, 85). For example, poly(GP) was detected in CSF of mice expressing an expanded *C9orf72* repeat in the brain, and CSF poly(GP) was decreased following treatment with a repeat RNA-targeting ASO. Of note, CSF poly(GP) levels correlated with DPR protein pathology, repeat RNA levels and RNA foci burden in the brains of mice (81). These data suggest that monitoring CSF poly(GP) before and during treatment of patients participating in clinical trials presents a feasible approach to gauge target engagement.

## Summary

The search for biomarkers of disease onset and progression in *C9orf72* repeat expansion carriers has yielded promising candidate biomarkers (Table 1). Clinically, cognitive, behavioral, and motor impairment occur on a continuum in patients with the *C9orf72* mutation. Non-invasive imaging studies in C9+ carriers have identified structural and functional changes in critical components of the networks associated with cognition and behavior. Early thalamic involvement has been detected in structural, functional, and metabolic imaging studies in C9+ carriers across different clinical phenotypes, in both prospective and retrospective studies. Diffusion changes in frontal white matter may also occur early in disease. These non-invasive imaging measures warrant further study in asymptomatic carriers as early markers of degeneration. Among the minimally invasive biomarker measures, CSF pNfH or NfL may allow identification of disease onset in asymptomatic carriers and forecast survival in symptomatic carriers (72, 77, 78). Now that *C9orf72* mutation carriers can be identified by genetic testing many decades before symptoms begin, and efforts to develop gene-directed therapy are underway, it is possible to imagine that biomarkers will play important roles in future therapeutic decisions. For example, in the future, persons known to carry the *C9orf72* mutation could undergo periodic screening with non-invasive tests such as MRI or physiology, followed by minimally invasive testing to measure CSF or blood biomarkers when findings suspicious for neurodegeneration arise.

**Table 1:** Timeframes for detecting changes in selected candidate biomarkers in *C9orf72* carriers.

	**Years prior to symptom onset**	**1 year prior to clinical symptoms**	**Early–mid stages of disease**	**Late stages of disease**
CSF dipeptide repeat proteins	•	•	•	•
Functional connectivity salience network (fMRI)	•	•	•	•
Thalamic atrophy	•	•	•	•
CSF NfL		•	•	?
Cortical hyperexcitability (TMS)			•	?
Reduced integrity of frontal white matter and association tracts (DTI)		?	•	•
CSF pNfH		?	•	•
FDG-PET frontotemporal hypometabolism			•	•
Global loss of functional connectivity			•	•
Global volume loss–ventricular atrophy, subcortical atrophy			•	•
Diffuse cortical thinning			•	•
Diffuse loss of white matter integrity (DTI)			•	•

*CSF, cerebrospinal fluid; DTI, diffusion tensor imaging; FDG-PET, fluoro-deoxyglucose proton emission tomography; fMRI, functional magnetic resonance imaging; pNfH, phosphorylated neurofilament heavy chain; NfL, Neurofilament light chain; TMS, transcranial magnetic stimulation. Question marks indicate measures needing further study*.

## Author Contributions

MF and TG drafted sections of the manuscript and proofread the entire manuscript.

### Conflict of Interest Statement

TG has a U.S. patent on methods and materials for detecting C9+ ALS and FTD using poly(GP). The remaining author declares that the research was conducted in the absence of any commercial or financial relationships that could be construed as a potential conflict of interest.
